# Delayed methimazole-induced agranulocytosis in a 6-year old patient with Graves’ disease

**DOI:** 10.1186/s13633-016-0034-6

**Published:** 2016-09-06

**Authors:** Vidya Puthenpura, Kinjal Desai, Andrew Bauer, Ian Marshall

**Affiliations:** 1 Department of Pediatrics, Rutgers-Robert Wood Johnson Medical School, 89 French Street, New Brunswick, NJ 08901 USA; 2 Division of Endocrinology and Diabetes, Children’s Hospital of Philadelphia, 324 S 34th St Ste 10, Philadelphia, PA 19104 USA

**Keywords:** Graves’ disease, Methimazole, Agranulocytosis, Absolute neutrophil count

## Abstract

**Background:**

Agranulocytosis is regarded as a rare side effect of methimazole (MMI) therapy that occurs in a dose dependent manner and that usually develops within the first 3–6 months of treatment. Although delayed development beyond this timeline has been documented in adults, very few children have been reported with this presentation.

**Case presentation:**

We present a 6-year old patient who developed agranulocytosis 18 months after the start of MMI therapy.

**Conclusions:**

This is an unusual case of a 6-year old patient who developed this serious side effect on stable MMI therapy well beyond the typical timeline. Our review of the literature revealed that there really is inconclusive data on the incidence, time, and dose-dependency of MMI-induced agranulocytosis in the pediatric Graves’ disease population.

## Background

Graves’ disease (GD) is the most common cause of hyperthyroidism in the pediatric population.[1] It generally presents as thyrotoxicosis, with a firm, nontender goiter, ophthalmopathy, peripheral tremor, tongue fasciculations, tachycardia, and/or hypertension on examination [2]. Diagnostic testing relies on confirmation of hyperthyroidism with elevated thyroid hormone and suppressed thyroid stimulating hormone (TSH) levels associated with the presence of TSH receptor antibodies (TSHR autoantibodies; TRAb), namely thyroid stimulating immunoglobulin (TSI) and TSH binding inhibitor immunoglobulins (TBII) [3].

Methimazole (MMI), a medication that inhibits thyroid peroxidase function, is currently the standard initial therapy for GD in the pediatric population [2]. MMI is associated with both minor and major side effects, the majority occurring within the first three months of initiating therapy [4]. Minor side effects include cutaneous eruptions and arthralgia, whereas major side effects consist of agranulocytosis, hepatotoxicity, and Steven Johnson syndrome [4].

We present a 6-year old patient with GD who developed severe agranulocytosis after 18 months on MMI therapy. We also present our review of the literature regarding the incidence, time, and dose-dependency of MMI-induced agranulocytosis in the pediatric GD population.

## Case presentation

The patient initially presented at 5 years of age to an outside hospital with a 1-week history of palpitations and chest pains. Physical examination revealed tachycardia, hypertension, tongue fasciculations, and a peripheral tremor. Initial testing confirmed primary hyperthyroidism with elevated free T4 level of 5.32 ng/mL (0.78–2.49; by immunoassay, Quest Diagnostics, Inc., NJ, USA) and suppressed TSH at <0.015 μIU/mL (0.460–8.100; by immunoassay, Quest Diagnostics, Inc., NJ, USA). TSI and TBII were elevated at 359 % (<140 %; by immunoassay, Quest Diagnostics, Inc., NJ, USA)) and 63.4 % (≤16 %; by radioreceptor assay, Quest Diagnostics, Inc., NJ, USA), respectively. Absolute neutrophil count (ANC) and liver transaminase levels were normal. She was diagnosed with GD by an outside pediatric endocrinologist and started on beta-blocker therapy for symptomatic relief, and MMI at an initial dose of 5 mg daily (~0.25 mg/kg/day). This dose was raised over the next 4 months up to 20 mg daily (~1 mg/kg/day). She continued on this dose with optimal thyroid function control. At age 5 ½ years, her family transferred care to our practice. They denied prior clinical evidence of agranulocytosis, including unexplained fevers, sore throat, and/or mouth sores. Subsequent routine testing revealed normal ANC between 1000 and 3300 cells/μL.

At a routine visit at age 6 ½ years, her family reported a 4-day history of a sore throat, and intermittent fevers, with 2 painful tongue lesions. There were no reported sick contacts, prior illnesses, or administration of other medications. Blood testing revealed ANC of 0 cells/μL. MMI therapy was immediately discontinued and she was admitted to the hospital for empiric antibiotic treatment. Her fevers resolved after 1 day. Her ANC remained at 0 cells/μL for the first 5 days and then increased to 100 cells/μL on day 6. Follow-up testing after 13 days off MMI showed ANC at 900 cells/μL.

Inpatient testing was within normal limits, including blood, urine, and throat cultures, and tongue lesion cultures for herpes simplex virus 1 and 2. Testing of liver enzymes, anti granulocyte antibodies, and IgM testing for Epstein Barr virus (EBV), parvovirus B19, and cytomegalovirus (CMV) was negative. The patient was assessed as having MMI-induced agranulocytosis based on negative testing for common viral causes of agranulocytosis, and improvement in ANC after discontinuation of MMI therapy.

The patient then underwent successful thyroidectomy surgery by an experienced pediatric surgeon without the development of any short and long-term complications. Her hypothyroidism is now well controlled on thyroxine replacement therapy.

## Discussion

MMI is the standard therapy for pediatric GD [2] with the goal of reaching a biochemical euthyroid state. RAI and thyroidectomy are typically reserved for children who do not achieve sustained remission on MMI and/or develop major side effects on MMI [1].

Propylthiouracil (PTU) was previously used until 2010 in the United States when the Food and Drug Administration issued a black box warning against its use in children [5]. This was based on reports of serious hepatotoxic effects of PTU in the pediatric population and that 30 % of PTU-related liver transplants between 1990 and 2007 were performed in children [6–8]. Furthermore, studies showed serial monitoring of transaminase levels was ineffective since PTU-induced hepatic injury had an insidious onset [7, 8]. These studies suggested that PTU was both specifically toxic in children and unpredictable with monitoring.

Side effects are still associated with MMI therapy. Minor effects, such as urticaria and arthralgia, affect up to 17 % of children on MMI therapy [4]. Major side effects are agranulocytosis, hepatotoxicity, and Stevens-Johnson syndrome [4]. Agranulocytosis, defined as ANC of ≤ 500 cells/μL [9], typically presents with fever, sore throat, and/or mouth sores [9]. The mechanism behind MMI-induced agranulocytosis is not fully understood, but thought to be the result of multiple factors, including direct drug toxicity, and immune-mediated destruction of mature granulocytes [9, 10]. A potential mechanism is that MMI and PTU act as haptens that trigger antibody formation against neutrophils and results in neutrophil destruction [10]. Another study demonstrated that antithyroid medications can easily penetrate bone marrow and affect oxygen and glucose utilization by neutrophils. This bone marrow microenvironment that is altered by the direct presence of the drug, negatively impacts differentiation of stem cells [11].

Although rare, the exact incidence of agranulocytosis in children specifically due to MMI therapy is unknown. The incidence of agranulocytosis due to both MMI and PTU in the pediatric population is 0.1 – 0.2 % [9]. We reviewed data from United States Food and Drug Administration Adverse Effects Reporting System (FAERS), a database of adverse event and medication error reports submitted to the FDA. Our review of all MMI-induced agranulocytosis cases reported over a 15-year period unfortunately revealed incomplete information, with crucial missing data that included patient age, MMI dose, and treatment duration.

The timing of the development of agranulocytosis after initiation of MMI therapy has been investigated. Various reports suggest that this most commonly develops within 6 months of starting therapy [8], with peak onset within 3 months [2]. However, 4 % of children develop adverse events including agranulocytosis much later at 18 months [12].

MMI-induced agranulocytosis has been suggested to be dose-dependent [2]. However, extensive review of literature revealed a paucity of data on the effect of the dose of MMI on development of agranulocytosis, particularly in the pediatric population. Takata et al. demonstrated a statistically significant 0.6 % higher prevalence of agranulocytosis in a cohort of both adult and pediatric patients treated with 30 mg MMI daily compared to 15 mg daily [13]. However, the results were not stratified according to age.

Our literature review could only identify 1 study by Minamitani et al. investigating dose-dependency of MMI-induced agranulocytosis in pediatric patients. In this retrospective review of 16 patients, with a median age of 13 years, the median time of agranulocytosis onset on 7.5–10 mg of MMI daily was 282 days after start of therapy. In contrast, patients on higher doses of 20–25 mg and 30–45 mg daily had shorter median onsets of 78 days and 35 days, respectively [9]. Additional studies with larger numbers of patients are necessary to assess true dose dependency of agranulocytosis.

The first step in treating MMI-induced agranulocytosis is immediate discontinuation of the drug. Since many studies have established a significant cross-reactivity between MMI and PTU, the use of this alternative antithyroid drug is contraindicated [9]. Initiation of intravenous broad spectrum antibiotics to cover Gram positive and negative, and anaerobic organisms is recommended, especially if the presentation includes fevers [14]. Although granulocyte-colony stimulating factor (G-CSF) therapy has been recommended to increase ANC and shorten recovery time, multiple studies have shown inconsistent results [15, 16].

Alternatives to MMI therapy are ^131^I radioactive iodine (RAI), and thyroidectomy [12]. Both should result in a chronic hypothyroid state, requiring exogenous thyroid hormone replacement. Increased risk of thyroid malignancy has been a concern of RAI therapy in pediatric GD. However, in more than 1200 children and adolescents treated with appropriate high doses of ^131^I for GD and followed from less than 5 to 15 years, with some for more than 20, increased risk of thyroid malignancy was not demonstrated [17]. The study by Read et al. with the longest follow-up of more than 100 children monitored for nearly 40 years after receiving ^131^I RAI, did not show adverse events or deaths that could be attributed to ^131^I therapy [18].

The oldest form of curative therapy in GD is thyroidectomy surgery [19]. When performed by an experienced pediatric surgeon at a high volume center, short and long-term complication rates, including hypoparathyroidism and recurrent laryngeal nerve injury, are very low [19].

## Conclusions

Our review of published data interestingly revealed inconclusive data about the incidence, time, and dose-dependency of MMI-induced agranulocytosis in the treatment of pediatric GD. It is therefore essential for all clinicians to continue to monitor for MMI-induced agranulocytosis regardless of duration, and dose of therapy. Further, as the presentation of agranulocytosis mimics common disease presentations, a high index of suspicion must be maintained in all patients on MMI therapy. Lastly, in the interests of collecting this crucial data on all side effects of MMI therapy, we strongly encourage all pediatric and adult endocrinologists to diligently utilize the FAERS reporting system (and in Europe, the equivalent EudraVigilance).

## Consent

Written informed consent was obtained from the patient’s legal guardian for publication of this case report. A copy of the written consent is available for review by the Editor-in-Chief of this journal.

## Abbreviations

ANC: absolute neutrophil count
CMV: Cytomegalovirus
EBV: Epstein barr virus
FAERS: FDA Adverse effects reporting system
G-CSF: granulocyte-colony stimulating factor
GD: Graves’ disease
MMI: Methimazole
PTU: Propylthiouracil
RAI: Radioactive iodine
TBII: TSH binding inhibitor immunoglobulins
TSH: Thyroid-stimulating hormone
TSHR autoantibodies; TRAb: TSH receptor antibodies
TSI: Thyroid-stimulating immunoglobulins
